# Examining the Effect of SNAP-Multibehaviours on Multimorbidity Risk: A Cross-Sectional Study in Three General Practices’ Electronic Health Records

**DOI:** 10.3390/ijerph22081251

**Published:** 2025-08-10

**Authors:** Konstantinos Spyropoulos, Naomi J. Ellis, Christopher J. Gidlow

**Affiliations:** 1 Centre for Health and Development (CHAD), University of Staffordshire, Stoke-on-Trent ST4 2DF, UK; n.j.ellis@staffs.ac.uk; 2 School of Medicine, Keele University, University Road, Staffordshire ST5 5BG, UK; c.gidlow@keele.ac.uk; 3 Research and Innovation Department, Midlands Partnership University NHS Foundation Trust (MPFT), St Georges Hospital, Corporation Street, Stafford ST16 3AG, UK

**Keywords:** multimorbidity, multibehaviours, multivariate analysis, primary care, sex specific multimorbidity patterns

## Abstract

Background: The growing challenge of multimorbidity for healthcare systems worldwide demands a dual prevention framework, targeting both primary and secondary prevention. Multimorbidity–multibehaviours can provide such a theoretical and clinical framework to explore new aetiological evidence for multimorbidity risk. While the role of single health risk behaviours, such as smoking, nutrition, alcohol, and physical activity (SNAP), in chronic disease prevention is well-documented, their synergistic effect on multimorbidity has received relatively little attention. Methods: Using retrospective observational data from electronic health records of 21,079 patients from a convenience sample of three general practices in Staffordshire, UK (2015–2018), we examined the association between SNAP-multibehaviours and multimorbidity risk, defined as follows: MM2+ (≥2 morbidities), MM3+ (≥3 morbidities), and complex multimorbidity (accumulated morbidities affecting ≥3 body systems). Multiple logistic regression models, stratified by sex and adjusted for age and area, were applied to analyse the associations between both combined and accumulative SNAP-health risk behaviours (HRBs) and all multimorbidity operational definitions. Results: A dose–response association was observed, indicating increased multimorbidity risk with greater accumulation of SNAP-HRBs. Additionally, sex-specific patterns were identified, which varied according to the operational definitions of multimorbidity. These findings underscored both the clinical significance of the identified outcomes for promoting tailored multimorbidity guidelines and the need for further sex-sensitive research. Conclusion: These findings support the importance of transcending traditional silos in healthcare and public health research by integrating preventive and curative medicines under a multimorbidity–multibehaviour framework. Embracing the complexity of coexisting morbidities and health risk behaviours, healthcare systems can move beyond disease-specific and behaviour-specific paradigms. This approach has the potential to enhance clinical outcomes and to address the complex needs of individuals with multimorbidity in real-world healthcare settings.

## 1. Introduction

Addressing multimorbidity (MM), the co-occurrence of two or more morbidities in the same person, requires a dual approach. Firstly, attention should be directed towards primary prevention to diminish the incidence of new cases of multimorbidity. Secondly, by halting progression and the associated complications of co-existing morbidities, secondary prevention plays a pivotal role in multimorbidity prognosis and optimal management of existing multimorbidity.

Health risk behaviours (HRBs) such as smoking, poor nutrition, excessive alcohol consumption, and physical (in)activity (SNAP) emerged in the multimorbidity literature as prime candidates for primary and secondary prevention, due to their extensively studied role in preventing the individual chronic conditions [1]. However, their association with multimorbidity began to receive attention approximately a decade ago [2], following calls from researchers for aetiological evidence of key determinants essential for the development of primary and secondary prevention measures [3].

Much of the research in this area has focused on examining single SNAP-HRBs and multimorbidity and generated mixed findings. For example, Fortin et al. [4] did not observe a protective association between physical activity and multimorbidity among males aged 65–94 years. On the contrary, Cimarras-Otal et al. [5] and Dhalwani et al. [6] both reported an inverse dose–response association. Inconsistent results were also found with alcohol binge drinking [7] and diet [8]. Despite findings on single SNAP-HRBs and multimorbidity risk, which may be influenced by methodological or statistical artifacts, they also emphasise a subtle pragmatic limitation—the oversight of the synergistic effects that accompany SNAP-HRBs clustering. A deeper understanding of these synergistic effects of SNAP-HRBs on multimorbidity risk is essential, requiring more nuanced analyses to yield more clinically meaningful insights. 

This includes recognising the increased likelihood of developing multimorbidity but also the potential for synergistic interventions targeting multiple SNAP-HRBs rather than individual behaviours [9]. Ultimately, this can inform the development of a multimorbidity–multibehaviours (MB) theoretical and clinical framework able to guide future research in this area [10].

This becomes particularly significant when considering the findings from Randell et al. [11] regarding primary care consultations in the UK. Their study indicated that 95.5% of those attending primary care were eligible for a health risk behavioural intervention, with almost half of them (43.6%) identified as suitable for a multibehavioural intervention.

However, few epidemiological studies have answered this call by exploring the combined and cumulative effect of SNAP-HRBs on multimorbidity risk. The association between multibehaviours varies depending on the number of SNAP-MB and the operational definition of multimorbidity applied. While social patterning issues for both multibehaviours and multimorbidity are well-acknowledged and investigated [12,13,14], there are several other issues accompanying them that remain poorly understood [15,16]. 

There is a need to better understand whether the commonly observed dose–response association between the number of SNAP-MB and multimorbidity risk indicates a pattern that transcends all multimorbidity operational definitions, rather than an artifact of simple counting measures of multimorbidity. Similarly, sex differences in multimorbidity risk remain puzzling. De Almeida et al. [17] found that all SNAP-HRBs were statistically significant predictors of multimorbidity in males but not in females when using the MM2+ definition (i.e., defining MM as having two or more conditions). Fortin et al. [4] reported that the risk of multimorbidity MM3+, (defined as three or more conditions) was significantly higher in males who engaged in all four SNAP-HRBs; for females, the risk was elevated with engagement in at least two SNAP-HRBs. Such evidence suggests a different threshold of developing multimorbidity between sexes, with males appearing to be primarily influenced by the number of SNAP-HRBs, whereas females’ risk seems more dependent on the multimorbidity definition.

This study aimed to comprehensively and comparatively examine the association between SNAP-MB and multimorbidity risk across multiple operational definitions (MM2+, MM3+, and complex multimorbidity), employing stricter cut-off points and stratified analyses by sex to further elucidate this complex relationship. 

## 2. Materials and Methods

### 2.1. Study Design and Processes

This retrospective observational study focused on multicentre data gathered between 2015 and 2018. The processes of data extraction and processing have been detailed previously [18]. The study examined the electronic health records (EHR) of a convenience sample of three general practices (GPs) in Staffordshire, UK, which used the EMIS web clinical system, minimising possible double counting and omissions and securing access to unadulterated data via EMIS standardised data coding (e.g., Read Codes).

NHS Midland and Lancashire Commissioning Support Unit (CSU) authorised specialised personnel were responsible for extracting the dataset and translating the Read Codes appropriately. A mock data extraction exercise was performed prior to the final extraction to ensure the accuracy of the extracted data. This applied methodology addresses concerns experienced by similar studies [19,20] regarding whether or not GP personnel have the experience to correctly use the technology accompanied by patients’ EHR records.

The extracted data concerned all those registered with the participatory GPs between 2015 and 2018 that were aged 18+ years. For each participant that met this criterion, three different types of information were extracted from their EHRs.

### 2.2. Sociodemographic Variables

Sociodemographic data were extracted for age, sex, ethnicity, and Lower Super Output Areas (LSOA) of residence. The LSOA allowed derivation of deprivation using the Index of Multiple Deprivation (IMD). The IMD serves as the official measure of relative deprivation in England and is integral to the suite of outputs comprising the Indices of Deprivation (IoD). Operating within an established methodological framework, the IMD broadly defines deprivation to encompass a wide range of an individual’s living conditions. IMD is ranked and used to derive deciles of deprivation, where 1 signifies the most deprived and 10 the least deprived. These deciles were then converted to quintiles for the present analyses, with Q1 representing the most deprived and Q5 the least deprived. 

### 2.3. Multimorbidity Index

As no standard approach exists regarding the measurement of multimorbidity [21,22], the current study’s methodology follows that applied by Barnett et al. [12] including a list of 40 physical and mental morbidities (Supplement file). This dual spectrum of number and type of morbidities met the minimum inclusion requirements posed by two systematic reviews [23,24] as the core for any multimorbidity measurement. According to them, any multimorbidity investigation should include at least 11 or 12, respectively, of the most common chronic conditions (cancer, diabetes, depression, hypertension, myocardial infarction, chronic ischemic heart disease, heart arrhythmia, heart insufficiency, stroke, COPD, arthritis) or among those that exist within the dataset, respectively.

Operationally, the two most used operational definitions (MM2+ and MM3+) were selected based on suggestions derived from the influential systematic review by Fortin et al. [24]. They recommended the cross-examination of both operational definitions, primarily due to the limited discriminatory ability of the more traditional MM2+ definition. 

We also adopted a complex multimorbidity definition, which determines whether a person has acquired three or more chronic conditions impacting at least three different organ systems. This was developed to increase the discriminatory value of multimorbidity operational definitions [14,25].

Huntley et al. [26], while assessing the predictive accuracy of the aforementioned traditional definitions (MM2+, MM3+) alongside complex multimorbidity, suggested an equally good performance. However, given the limited application of complex multimorbidity definition within the literature, they recommended the combined implementation of all three definitions to increase the validity of the identified outcomes. Following this recommendation, the present study applied all three operational definitions of multimorbidity.

Furthermore, with the assistance of CSU personnel, the list of the 40 morbidities from Barnett et al.’s [12] multimorbidity index has been adjusted to Cumulative Illness Rating Scale (CIRS) body systems, including cardiovascular, respiratory, visual, cancer, hepatic, gastrointestinal, mental, neurological, endocrine, sensory, renal, and musculoskeletal.

### 2.4. Multibehaviours

Information was extracted that related to the following four most common health risk behaviours (HRBs): smoking, nutrition, alcohol, and physical activity (SNAP). To obtain the most accurate picture regarding patient’s involvement with the SNAP-HRBs and acknowledging the limitations around recording of this information in primary care, the EHRs were used to extract information based on the measurement of the behaviours (if present) and evidence of patients being given advice relating to changing these behaviours. These are detailed in turn:

- Smoking status was extracted from EHRs as ‘current smokers’, ex-smokers’, and ‘never-smokers’. For pragmatic and theoretical reasons, these were regrouped into a binary categorisation as ‘ever-smoker’ and ‘never-smoker’. Practically, it was expected that the binary categorisation better captures the cumulative smoking exposure over time, which may be more relevant for assessing its association with multimorbidity than current or former status alone, and it would better facilitate the examination of associations of combined and accumulative SNAP-HRBs with multimorbidity risk. Additionally, many epidemiological studies examining the association between smoking and multimorbidity have used binary smoking categories [27]. Methodologically, it is expected that binary categorisation enhances the statistical power to detect significant associations between smoking status and multimorbidity and helps to mitigate potential misclassification biases that may have been introduced to the system via the registration process and associated with self-reported smoking status, which may vary in accuracy across different population groups [28]. Healthcare providers’ advice, such as ‘health education’ or ‘smoking cessation advice,’ were categorised as ‘ever-smoker’ in binary coding.
- Nutrition was categorised as a poor diet (meaning lack of regular fruits/vegetables per day and/or fat unhealthy diet), average diet (diet that has periodically both the characteristics of unhealthy and healthy diet), and healthy diet (that meets both the criteria of low-fat diet rich in vegetables and fruits). Again, for practical and statistical consistency, binary coding was applied to diet classifications. ‘Poor’ and ‘average’ diets were recorded as ‘bad nutrition,’ while ‘good’ diets remained unchanged. Healthcare providers’ advice was also considered. For example, recommendations such as ‘patient advice about weight-reducing diet,’ ‘healthy eating advice,’ and ‘patient advice for low-cholesterol diet’ were all categorized as bad nutrition.
- Alcohol intake was based on the consumption of alcohol units per week. As such, it was classified as ‘excessive alcohol usage’ when alcohol intake was greater than the 14 units per week, ‘normal drinking consumption’ when it did not exceed the 14 units per week, or ‘never drinking’. The binary coding for this category involved recording ‘normal drinking consumption’ and ‘never drinking’ as ‘normal drinking,’ while excessive alcohol usage remained unchanged. Healthcare providers’ advice, such as ‘advice on alcohol consumption,’ ‘lifestyle advice regarding alcohol,’ or ‘alcohol health promotion,’ among others, were all recorded as excessive alcohol usage.
- Physical activity was classified based on the guidelines of 150 min of moderate activity or 60 min of vigorous activity per week. Binary coding was conducted as follows: individuals initially classified as ‘moderately active’ or ‘inactive’ were recorded as physically inactive’, while those originally labelled ‘active’ or ‘meeting the recommended guidelines,’ remained unchanged. Healthcare providers’ advice, such as ‘health education—exercise’ or ‘patient advice about exercise,’ were all coded as physically inactive.

For decoding suggestions based on a Read Code system, an assistant from the CSU team and other health specialists such as a dietitian with a PhD was obtained. Extracted data were anonymous, and as such, no possible identification of participants was possible. Ethical approval was obtained from the NHS Health Research Authority (East of England, Essex Research Ethics Committee).

### 2.5. Statistical Analysis

Frequency calculations provided a descriptive analysis of characteristics of the study population, an estimation of the prevalence of all types of multimorbidity, the various morbidities included in multimorbidity measurement, as well as all SNAP-HRBs, single or combined. Chi-square analyses were used to examine the association between multimorbidity and SNAP-HRBs associations with sociodemographic variables, such as age, sex, and deprivation (possible confounders of the MM-SNAP association). Sequentially, multiple logistic regression models assessed the odds of acquiring multimorbidity, using each of the multimorbidity definitions, by engagement with any combined, accumulative SNAP-HRBs. This was explored through unadjusted and adjusted models and stratified by the sociodemographic covariate of sex. Other types of stratified analyses, such as deprivation, were not undertaken. The decision was made solely based on methodological considerations. Although all relevant data were extracted and prepared for analysis, factors such as the heightened risk of reverse causality outcomes in the association between multimorbidity, multibehaviours, and the geographical area of residence, stemming from a highly skewed cohort, deterred the execution of specific analyses.

Finally, multiple imputation was applied to address the missingness problem, overcoming the biases possible when a missing value(s) are detected (and cases excluded from analysis). IBM-SPSS (version 28, Chicago, IL, USA) was used for data preparation and analyses.

## 3. Results

### 3.1. Addressing the Issues of Missing Data

The percentage of missing values ranged from zero (all variables that represent the included morbidities) up to 55.6% (nutrition variable). The rest examining SNAP-HRBs such as smoking, alcohol, and exercise had missing values of 7.6%, 26.1%, and 39.3%, respectively. For the demographic variables, these ranged from almost zero for deprivation to 23.4% for ethnicity, reaching the highest missing rate of 85.6% for employment variable to a sample of 21,079 participants.

Given that variables with most missing data were demographic issues like employment and SNAP-HRBs, but not patients’ disorders, it is possible that there are inconsistencies in procedures during the registration of patients at general practices. Other reasons may be the reluctance of patients to share sensitive personal information (e.g., the amount of daily alcohol drinking) or lack of thorough follow up regarding the inclusion of adherence to SNAP guidelines.

The issue of missing data was addressed using multiple imputation in SPPS. Specifically, by visually examining the existence or not of monotonicity in missing data (by inspecting the appearance of specific pattern), confidence was gained that values were missing at random, since no such pattern revealed. To achieve the best possible imputed value outcomes, all auxiliary variables were included within multiple imputation. As such, a Markov Chain Monte Carlo method by a logistic regression model was applied, since no monotonicity was found, and all included variables were categorical ones. Indicating 10 iterations for this process, SPSS generated five imputed datasets, whereby applying “Rubin’s rules” a pooled dataset was produced [29,30]. Running a logistic regression to four SNAP-HRBs, a reasonable comparison between imputed and observed values was implemented. All statistical analyses were performed on the pooled imputed dataset.

### 3.2. Sociodemographic Characteristics

Table 1 summarises the study population characteristics. Sex distribution showed similar proportions of males and females (52.1% and 47.9%, respectively). The rest of the sociodemographic variables, when measured, were highly skewed. The majority of the sample were classified as British/mixed British or White (84.7%), with those being classified as Arab, Asian, and Black accounting for much smaller proportions (8.85%, 3.8%, and 2.7% respectively). The younger age group of 18–45 was overrepresented, comprising almost half the study population (48.7%). Approximately one-third were 46–66 years old (32.1%), and 19.2% were age 67 years old or more. The age cut offs applied followed those regularly appearing in multimorbidity studies [31,32].

In relation to SNAP-HRBs, only 0.4% of the total group of participants did not engage with any of the four SNAP-HRBs. Excess alcohol intake was the most common behavioural risk factor (92.3%). Poor nutrition and those who had ever-smoked (smokers and ex-smokers) followed with 40.8% and 38.5%, respectively, while physical inactivity appeared with much lower rates of 18.6%. Multiple SNAP-HRBs reached 92% with only 7.6% of the study’s population engaging with a single SNAP-HRB. The prevalence of the most often applied multimorbidity operational definitions were 38.3% for MM2+, 23.5% MM3+, and 19.1% for complex MM. Finally, the mean number of chronic conditions was 3.06 (SD = 1.75).

Chi squares analyses showed that all single SNAP-HRBs (χ^2^ = 402.46 (2), *p* < 0.001; χ^2^ = 784.17 (4), *p* < 0.001; χ^2^ = 1304.64 (8), *p* < 0.001 smoking; χ^2^ = 597.07 (2), *p* < 0.001; χ^2^ = 1055.98 (4), *p* < 0.001; χ^2^ = 2984.23 (8), *p* < 0.001 nutrition; χ^2^ = 27.42 (2), *p* < 0.001; χ^2^ = 133.15 (4), *p* < 0.001; χ^2^ = 87.06 (8), *p* < 0.001 alcohol; χ^2^ = 114.84 (3), *p* < 0.001; χ^2^ = 411.60 (6), *p* < 0.001; χ^2^ = 896.72 (12), *p* < 0.001 physical activity) were significantly associated with sex, age, and deprivation (Table 2). 

Similar significant associations were also observed between the sociodemographic variables and all multimorbidity operational definitions (χ^2^ = 275.33 (1), *p* < 0.001; χ^2^ = 4157.26 (2), *p* < 0.001; χ^2^ = 141.21 (4), *p* < 0.001 MM2+; χ^2^ = 156.26 (1), *p* < 0.001; χ^2^ = 4298.82 (2), *p* < 0.001; χ^2^ = 130.55 (4), *p* < 0.001 MM3+ and χ^2^ = 101.78 (1), *p* < 0.001; χ^2^ = 4361.39 (2), *p* < 0.001; χ^2^ = 109.11 (4), *p* < 0.001 complex MM).

### 3.3. Combined SNAP-HRBs - Overall

The outcomes of regression models and of dual combinations of SNAP-HRBs before and after adjustment for age, sex, and deprivation are presented in Table 3. In short, when adjusted, all SNAP-HRB combinations were significantly associated with all types of multimorbidity operational definitions, ranging from 15% higher odds of developing MM2+ if smoking combined with alcohol usage, to 75% increased odds of developing complex MM when poor nutrition is combined with excessive alcohol usage. Generally, nutrition was found to be the key component of the most significant combined SNAP-HRB associations for all multimorbidity definitions.

As such, for MM2+, the combination of nutrition–alcohol produced the higher outcome effects with 38% increased odds (adj. OR = 1.38 95%CI:12.9–1.47) of multimorbidity compared to those not engaging in any of the SNAP-HRBs, followed by the smoking–nutrition combination with 27%. Smoking–nutrition was the most significant combination for MM3+, with a marginally stronger association than the nutrition–alcohol combination (54% and 53%, respectively). For complex MM, nutrition–alcohol again produced the highest outcome effect for multimorbidity risk with 62% odds of multimorbidity, followed by the nutrition–physical inactivity combination (60%).

### 3.4. Combined SNAP-HRBs - Stratified Analyses by Sex

Despite females showing stronger associations than males for most of the combined SNAP-HRBs (Table 4), when stratifying analyses by sex and adjusting for age and areas of living, a consistent pattern was observed only for MM2+ and complex MM. For these two multimorbidity definitions, the only combination that produced an outcome effect that was higher for males than the females was physical activity–alcohol. Specifically, for MM2+, the effect was 15% higher in males (adj. OR = 1.23 95%CI 1.12–1.36) than females (adj. OR = 1.19 95%CI 1.08–1.30), while for complex MM, the effect was 28% higher (adj. OR = 1.60 95%CI 1.41–1.82) in males, with 60% increased odds of developing multimorbidity versus the 32% increased odds in females (adj. OR = 1.32 95%CI: 1.18–1.48) comparing with their counterparts who do not engage with any SNAP-HRBs. The remainder showed more significant associations for females, with differential effects ranging from 5% to 20% under MM2+ and from 2% to 27% for complex MM, both for smoking–alcohol and smoking–nutrition combinations, respectively. For MM3+, the combined SNAP-HRB associations produced mixed results. Indicatively, three combinations of smoking–alcohol (adj. OR = 1.40 95%CI 1.25–1.57 males VS adj. OR = 1.38 95%CI 1.24–1.54 females), smoking–physical (in)activity (adj. OR = 1.52 95%CI 1.35–1.71 males VS adj. OR = 1.47 95%CI 1.31–1.65 females), and physical (in)activity–alcohol (adj. OR = 1.49 95%CI 1.33–1.68 males VS adj. OR = 1.23 95%CI 1.11–1.37 females) were more strongly associated with multimorbidity in males than females. While the remaining three combinations of smoking–nutrition (adj. OR = 1.45 95%CI 1.29–1.63 males VS adj. OR = 1.69 95%CI 1.50–1.91 females), nutrition–physical activity (adj. OR = 1.42 95%CI 1.27–1.59 males VS adj. OR = 1.54 95%CI 1.40–1.70 females), and nutrition–alcohol (adj. OR = 1.52 95%CI 1.34–1.72 males VS adj. OR = 1.60 95%CI 1.45–1.77 females) had associations that were stronger in females than in males.

Another sex discrepancy was observed in relation to the most significant combined SNAP-HRBs. Nutrition–alcohol combination was the most important for both sexes, with all multimorbidity definitions. In females, nutrition remained the common dominator, combined with smoking, producing the second highest odds of multimorbidity risk. While for males, physical (in)activity and alcohol had the second strongest association with MM risk.

### 3.5. Combined SNAP-HRBs - Accumulative HRBs

Accumulation of SNAP-HRBs in one person showed a positive dose–response association between the number of SNAP-HRBs and multimorbidity risk, which became stronger when the same number of SNAP-HRBs were examined under the MM2+, MM3+, and complex MM (Table 5). As such, the effect of any two SNAP-HRBs for complex MM (adj. OR = 1.50, 95%CI 1.33–1.69) was higher than the one for MM3+ (adj. OR = 1.48, 95%CI 1.33–1.65), which in turn, was higher than the one for MM2+ (adj. OR = 1.34, 95%CI 1.22–1.47).

The same pattern was identified for the associations of any three or four SNAP- HRBs (adj. OR = 2.17, 95%CI 1.89–2.4), (adj. OR = 2.10, 95%CI 1.85–2.38), (adj. OR = 1.57, 95%CI 1.42–1.73) for complex MM, MM3+, and MM2+, respectively.

### 3.6. Stratified Analyses by Sex

A clear dose–response association within a group and a positive gradient of outcome effect towards males emerged when analyses were stratified by sex and adjusted for age and deprivation (Table 6). Males had 18%, 31%, and 32% higher risk of multimorbidity than females when engaging in any two SNAP-HRBs investigated under the MM2+, MM3+, and complex MM, respectively. Despite the range of differences in outcome effects between sexes decreasing to 4%, 22%, and 23% with any three or four SNAP examined at MM2+, MM3+, and complex MM, they remained significant between the sexes.

## 4. Discussion

### 4.1. Main Findings

Given the lack of a standardised approach to measuring multimorbidity risk, we assessed the combined effect and accumulated associations of SNAP-HRBs alongside traditional simple count measurements (MM2+, MM3+) and the alternative operational definition, complex multimorbidity, as examined through the CIRS cumulative index. This comprehensive approach, while controlling for sociodemographic variables, was important to achieve more robustly validated outcomes, as highlighted by Huntley et al. [26]. Stratification of analysis by sex shed light on an issue that has puzzled the multimorbidity inquiry since its inception providing both clinically and theoretically valuable insights.

Our analyses identified the following three main findings: the importance of all forms (combined and accumulative) of multiple SNAP-HRBs to multimorbidity risk, in relation to all applied multimorbidity operational definitions, as reflected by their strong statistically significant outcome effects; dose–response associations for most of the interrelations between multiple SNAP-HRBs and multimorbidity, with all applied multimorbidity operational definitions; sex-specific patterns, which varied according to the operational definitions of multimorbidity.

The present study confirms and extends the evidence of newly emerging literature regarding the interrelationship between multibehaviours and multimorbidity, by demonstrating that all forms of multiple SNAP-HRBs are associated with the increased risk of multimorbidity, regardless of the operational definition. The main evidence extracted from current analysis was that whether combined (specific dyads) or accumulated (any form of two, three, or all four), SNAP-HRBs significantly predicted multimorbidity for all applied types of multimorbidity operational definition (MM2+, MM3+, complex MM), with a positive dose–response association. The only exception was the smoking–alcohol combination, which only produced a dose–response effect for complex MM (marginal for other MM definitions). 

Many researchers have also observed such a dose–response association. For example, Adams et al. [21], Dhalwani et al. [1], Fortin et al. [4], and Katikireddi et al. [33] all found a significant increase in odds for developing multimorbidity with the addition of a SNAP-HRB. Loprinzi [10] showed the existence of an inverse dose–response association, as a preventive mechanism to developing multimorbidity, when examining the accumulative health enhancing properties of the following three HRBs: no smoking, being physical active, and having a healthy diet.

The present findings have shown that all six possible SNAP-HRB combinations generated significant outcome effects in relation to multimorbidity risk. This evidence challenges the outcomes of other studies that have also examined specific SNAP dyads. Loprinzi et al. [10], for example, found significant predictive outcomes only when physical activity was combined with nutrition or smoking, and only for males. No significant association was identified for females for any possible SNAP combination. In the same trend, Dhalwani et al. [1] found significant predictive outcomes only for smoking when combined with physical activity or nutrition but failed to identify any significance for alcohol when combined with physical activity or smoking. Greater consistency was observed when the accumulated associations of SNAP-HRBs with multimorbidity risk were examined. The findings presented here align with the studies of Agrawal et al. [34], Adams et al. [21], and Katikireddi et al. [33], who all found significant effects and subsequent dose–response associations between any type of accumulated SNAP-HRBs (e.g., two, three, four) and the development of MM2+. There was no harmonization, however, with the results of Fortin et al. [4] for MM3+, where a threshold of two SNAP-HRBs in females and a corresponding threshold of four SNAPs-HRBs in males was needed before a significant association with multimorbidity risk was observed. Shao et al. [35], on the other hand, confirmed the present study’s results, verifying the existence of a significant associations of all types of accumulative SNAP-HRBs alongside a dose–response effect. Finally, the identification of the higher effect of accumulative SNAP-HRBs on complex MM risk reported here has no equivalent in literature. Nevertheless, Shao et al. [35] reported a significant association for all aggregated SNAP-HRBs on MM4+, which is a similarly stricter criterion, compared with the more commonly used multimorbidity operational definition MM2+ and MM3+.

All combined dyads of SNAP-HRBs were found to be important predictors of multimorbidity risk for all the applied operational multimorbidity definitions. The association regarding multimorbidity risk was higher for females than for males for all dyads within MM2+ and complex MM apart from the physical activity–alcohol combination (where associations were stronger for males). As for MM3+, findings were similar for both sexes. Females showed higher effects for all combined forms of nutrition, while in males, stronger associations were observed with smoking–alcohol, smoking–physical activity, and physical activity–alcohol SNAP dyads.

Regarding the accumulation of SNAP-HRB and multimorbidity risk, a sex pattern was observed but with stronger associations for males than with females for all forms of SNAP-HRB accumulations and under all multimorbidity definitions. This evidence has no equivalent in literature, since no sex pattern has been found by any other study that examined the same parameters. Indicatively, Fortin et al. [4] found that MM3+ risk was significantly predicted when females had at least two SNAP-HRBs and males engaged in all four SNAP-HRBs.

### 4.2. Implications for Research and Practice

Generally, the findings of the present study answer the call for aetiological evidence on multimorbidity development [2,32]. The complexity that emerges from the interrelations between all types of multimorbidity (MM2+, MM3+, complex MM) and all forms of multiple SNAP-HRBs (combined, accumulative) provides further support for a more holistic management of care that extends beyond the current medically monomorbid system. A basic characteristic of such a healthcare system will be the coalition of preventive and curative medicine toward a unified multibehaviours–multimorbidity theoretical and clinical framework [10]. Via the synthesis of interdisciplinary evidence, clinically valuable knowledge will emerge, offering a new mode of explanation to address the complexity posed by the new normalities of multibehaviours and multimorbidity. In turn, we will be able to address more efficiently the complex needs of people with multimorbidity.

Several challenges will accompany this effort. One of the most crucial challenges is to better align theory with reality. It could be argued that while behavioural and clinical–epidemiological researchers thrive within their scientific specialties, the mainstream of articles and studies continues to examine the effects of single behaviours and/or single diseases. People experience the complexities of multibehaviour–multimorbidity and their interaction with the present provision of preventive and curative care. In 2008, Prochaska [36] argued for the need to break down the disciplinary clinical and academic silos as a prerequisite to effectively face the current challenges. This challenge remains. For example, instead of debating whether a single or multiple health behavioural change (MBHC) intervention is more effective [37], it could be more pragmatic to seek a consecutively or congruent MBCH intervention that is more appropriate for people with multimorbidity [10,21], taking into consideration both the demands of everyday condition management [38] and the severe time constraints faced by general practitioners/healthcare staff, limiting further the effectiveness of MBCH interventions [36]. In the same spirit, policymakers must promote more collaborative and, where possible, interdisciplinary models of care, applying evaluation protocols to ensure that people with complex multimorbidity are supported more holistically. Training and resource allocation to healthcare personnel in primary care will not only increase the healthcare sector’s capacity and capability but will also support more effectively the engagement of people with multimorbidity in more meaningful discussions regarding lifestyle changes as effective management of MM. 

### 4.3. Limitations and Strengths of the Study

This is the first epidemiological study to comprehensively analyse both traditional count measurements (MM2+ and MM3+) alongside complex multimorbidity in a multimorbidity–multibehaviours inquiry. This synthesis not only enhances the validity of the study’s outcomes, as demonstrated by Huntley et al. [26] and Fortin et al. [4], but also fosters deeper understanding. It underscores the importance of adopting an approach that is both clinically practical and policy relevant, aligning with the principles advocated by Prochaska [36].

Study limitations are recognised. First, the cross-sectional design precludes the investigation of the temporal sequence of the interrelation between SNAP-HRBs and the development of multimorbidity, and design prohibits any inferences regarding causality. Second, the collection of data, especially SNAP-HRBs data, may have resulted in some misclassifications due to the well-known weaknesses of the registration system and routine collection (or lack of) of such data within the general practices. For example, people may hide specific issues that they deem embarrassing, or others may be under- or overestimated. Other misinformation may exist because people cannot perfectly define or recall the duration of their engagement with specific health behaviour (e.g., physical activity). Finally, general practitioners’ suggestions for lifestyle changes that were also used to define HRBs in the present study may have led to some misclassification.

While multiple imputations have been applied to address these issues, they should not be perceived as an absolute remedy, and caution is suggested when interpreting statistical results and their generalisation. Simulation studies [39] have shown that missing data, even when multiple imputed, can lead to biased estimations, as the consistency that may follow multiple imputation models can still produce datasets that do not accurately reflect the characteristics of the population under investigation.

Finally, the use of a convenience sample constrains further the generalisability of the study’s results, due to the potential sampling bias that may result. Such limitations on generalisability may be related to sample characteristics that deviate from those of the population under study. Geographical characteristics related to the allocation of general practices, whether in rural or urban territories, may also distort the demographic diversity of the sample. Individually or in conjunction, such methodological issues can affect the external validity of the present study. 

Future studies should be designed to address these limitations. Cluster randomized studies with longitudinal designs could help alleviate many of the aforementioned methodological barriers, reducing, for example, possible selection biases and/or the deficiencies of cross-sectional snapshots.

The strengths of the study include the large sample size, the multimorbidity index used that exceeds the minimum limit of including the 12 most important chronic conditions, use of electronic health records for extracting participants morbidities, and the implementation of multiple imputation.

## 5. Conclusions

The high prevalence of all multimorbidity types (MM2+, MM3+, complex MM) suggests the need to shift toward a more holistic approach to care, beyond the management of single disease. Yet, their significant associations with all forms of multiple SNAP-HRBs (combined and accumulative) produce a complex situation that requires a shift of the entire healthcare system paradigm and the reorientation of its priorities, goals, and targets. A basic characteristic of such a system will be its person- rather than disease-focus. Preventive and curative medicine should align toward the healthcare systems’ overarching goals, breaking down disciplinary silos, and via the creation of multidisciplinary teams, addressing the complex needs of patients with multimorbidity.

## Figures and Tables

**Table 1:** Sociodemographic characteristics of participants.

Groups	N	%	95%CI	
			Lower	Upper
Sex	21,079			
Males	10,986	52.1	51.4	52.8
Females	10,093	47.9	47.2	48.6
Age groups				
18–45	10,258	48.7	48	49.4
46–66	6773	32.1	31.5	32.7
67+	4048	19.2	18.7	19.7
Ethnicity				
White	8821	41.8	41.1	42.5
Mixed	9033	42.9	42.2	43.6
Asian	803	3.8	3.5	4.1
Black	566	2.7	2.5	2.9
Arabs/other	1856	8.8	8.4	9.2
Area of living				
Most deprived	3367	16.0	15.5	16.4
Deprived	2674	12.7	12.2	13.1
Moderately deprived/affluent	2423	11.5	11.0	11.9
Affluent	3905	18.5	17.9	19.0
Most affluent	8710	41.3	40.6	41.9
HRBs				
0 HRB	81	0.4	0.3	0.6
ANY SNAP-HRB	20,998	99.6	99.4	99.6
SNAP-HRB 1	1608	7.6	7.2	7.9
SNAP-HRB 2	6114	29	28.3	29.6
SNAP-HRB 3	9592	45.5	44.8	46.1
SNAP-HRB 4	3684	17.5	16.9	18.0
Smoking				
Smoker	5008	23.8	23.2	24.4
Ex-smoker	3105	14.7	14.2	15.2
Non-smoker	12,966	61.5	60.8	62.2
Alcohol				
Excessive	19,463	92.3	91.9	92.7
Normal	734	3.5	3.3	3.7
Never	882	4.2	3.9	4.5
Physical Activity				
Inactive	3930	18.6	18.1	19.1
Moderate inactive	3241	15.4	14.9	15.9
Moderately active	7125	33.8	33.2	34.4
Active	6783	32.2	31.6	32.8
Nutrition				
Poor diet	8609	40.8	40.1	41.5
Average diet	6133	29.1	28.5	29.7
Heathy diet	6337	30.1	29.5	30.7
Morbidities				
Atrial fibrillation	452	2.1	1.9	2.2
Heart failure	202	1.0	0.8	1.1
Hypertension	3821	18.1	17	18
Peripheral vascular disease	171	0.8	0.67	0.92
Stroke and& transient ischemic attack	455	2.2	2	2.3
Coronary heart disease	721	3.4	3.1	3.6
Asthma	2542	12.1	11	12
Bronchiectasis	94	0.4	0.3	0.4
Chronic sinusitis	255	1.2	1.01	1.3
Chronic obstructive pulmonary disease	400	1.9	1.7	2
Blindness	137	0.6	0.4	0.7
Glaucoma	456	2.2	2	2.3
Cancer	427	2.0	1.8	2.1
Prostate disorders	463	2.2	2	2.3
Chronic liver disease	336	1.6	1.4	1.7
Constipation	409	1.9	1.7	2.08
Diverticular disease	460	2.2	2	2.3
Dyspepsia	4026	19.1	18.5	19.6
Inflammatory bowel disease	1356	6.4	6.06	6.73
Irritable Bowel Syndrome	1340	6.4	6.06	6.73
Alcohol problems	276	1.3	1.14	1.45
Anorexia or bulimia	49	0.2	0.13	0.26
Anxiety	1571	7.5	7.14	7.85
Dementia	179	0.8	0.67	0.92
Depression	2727	12.9	12.44	13.35
Schizophrenia	179	0.8	0.679	0.92
Epilepsy	211	1.0	0.86	1.13
Migraine	236	1.1	0.95	1.24
Multiple Sclerosis	61	0.3	0.22	0.37
Parkinsons disease	64	0.3	0.22	0.37
Diabetes	1260	6.0	5.679	6.32
Hearing loss	2304	10.1	10.47	11.32
Chronic Kidney Disease	655	3.1	2.86	3.33
Painful condition	1688	8.0	7.63	8.36
Psoriasis/eczema	418	2.0	1.81	2.18
Rheumatoid arthritis	186	0.9	0.77	1.027
Thyroid	1239	5.9	5.58	6.21
Number of morbidities				
0	9284	44.0	43.36	44.71
1	3719	17.6	17.12	18.15
2	3121	14.8	14.32	15.27
3	1988	9.4	9.03	9.82
4	1187	5.6	5.31	5.94
5	734	3.5	3.23	3.72
6	470	2.2	2.02	2.41
7	251	1.2	1.04	1.33
8	153	0.7	0.6	0.83
9	95	0.5	0.35	0.54
10	39	0.2	0.12	0.23
11	24	0.1	-0.6	0.15
12	10	0.0	-0.1	0.7
13	3	0.0	-0.1	2.9
14	1	0.0	-0.4	1.2
Multimorbidity definition				
CC	9284	44.0	43.3	44.6
MM2+	8076	38.3	37.6	39
MM3+	4955	23.5	22.9	24.1
Cmpx MM	4025	19.1	18.4	19.5

HRB = health risk behaviours; SNAP = smoking, nutrition, alcohol, physical activity; CC = chronic condition; MM+2 = multimorbidity of 2+CC; MM+3 = multimorbidity of 3+CC; Cmpx MM = complex multimorbidity.

**Table 2:** Associations between sociodemographic variables, SNAP-HRBs, and multimorbidity operational definitions.

	Sex			Age			Area of Living		
	χ^2^	df	*p* Value	χ^2^	df	*p* Value	χ^2^	df	*p* Value
Smoking	**402.46**	2	*p* < 0.001	**784.171**	4	*p* < 0.001	**1304.648**	8	*p* < 0.001
Nutrition	**597.074**	2	*p* < 0.001	**1055.984**	4	*p* < 0.001	**2984.235**	8	*p* < 0.001
Alcohol	**27.424**	2	*p* < 0.001	**133.15**	4	*p* < 0.001	**87.064**	8	*p* < 0.001
Physical activity	**114.845**	3	*p* < 0.001	**411.601**	6	*p* < 0.001	**896.726**	12	*p* < 0.001
MM2+	**275.336**	1	*p* < 0.001	**4157.263**	2	*p* < 0.001	**141.215**	4	*p* < 0.001
MM3+	**156.268**	1	*p* < 0.001	**4298.82**	2	*p* < 0.001	**130.555**	4	*p* < 0.001
Complex MM	**101.784**	1	*p* < 0.001	**4361.397**	2	*p* < 0.001	**109.114**	4	*p* < 0.001

Bold denotes the statistical significance of the outcome.

**Table 3:** Unadjusted and adjusted odds ratios for incident multimorbidity by combined SNAP-HRBs.

SNAP-HRBs Combined	Unadjusted OR (95%CI)	Adjusted OR (95%CI) by Age, Sex, and IMD
MM2+		
Smoking–Alcohol	1.03 (0.97–1.09)	**1.15 (1.08–1.23)**
Smoking–Nutrition	**1.08 (1.02–1.15)**	**1.27 (1.18–1.37)**
Smoking–P.A.	**1.24 (1.16 (1.32)**	**1.23 (1.14–1.33)**
Nutrition–P.A.	**1.36 (1.28–1.44)**	**1.26 (1.19–1.35)**
Nutrition–Alcohol	**1.35 (1.28–1.44)**	**1.38 (1.29–1.47)**
P.A.–Alcohol	**1.55 (1.47–1.65)**	**1.21 (1.13–1.29)**
MM3+		
Smoking–Alcohol	**1.21 (1.14–1.30)**	**1.39 (1.29–1.50)**
Smoking–Nutrition	**1.28 (1.19–1.37)**	**1.54 (1.42–1.68)**
Smoking–P.A.	**1.49 (1.39–1.60)**	**1.50 (1.38–1.63)**
Nutrition–P.A.	**1.57 (1.48–1.68)**	**1.47 (1.37–1.58)**
Nutrition–Alcohol	**1.52 (1.41–1.63)**	**1.53 (1.42–1.66)**
P.A.–Alcohol	**1.78 (1.66–1.91)**	**1.34 (1.24–1.45)**
Complex MM		
Smoking–Alcohol	**1.21 (1.13–1.30)**	**1.36 (1.25–1.48)**
Smoking–Nutrition	**1.31 (1.21–1.41)**	**1.57 (1.43–1.72)**
Smoking–P.A.	**1.53 (1.42–1.65)**	**1.52 (1.39–1.66)**
Nutrition–P.A.	**1.70 (1.59–1.82)**	**1.60 (1.47–1.73)**
Nutrition–Alcohol	**1.60 (1.48–1.73)**	**1.62 (1.49–1.77)**
P.A.–Alcohol	**1.94 (1.79–2.09)**	**1.44 (1.32–1.57)**

IMD = Index of multiple deprivation, P.A. = Physical Activity; Emboldened text signifies statistical significance.

**Table 4:** Unadjusted and adjusted odds ratios for incident multimorbidity by combined SNAP-HRBs stratified by sex.

	Odds Ratios for Incident Multimorbidity by Combined SNAP-HRBs Stratified by Sex			
SNAP-HRBs Combined	Unadjusted OR (95%CI)		Adjusted OR (95%CI) by Age, Sex, and IMD	
	Male	Female	Male	Female
MM2+				
Smoking–Alcohol	**1.09 (1.00–1.18)**	**1.12 (1.03–1.23)**	**1.13 (1.02–1.24)**	**1.18 (1.07–1.31)**
Smoking–Nutrition	**1.09 (1.00–1.18)**	**1.34 (1.21–1.48)**	**1.20 (1.09–1.33)**	**1.40 (1.25–1.56)**
Smoking–P.A.	**1.31 (1.21–1.43)**	**1.29 (1.17–1.42)**	**1.19 (1.08–1.32)**	**1.27 (1.14–1.41)**
Nutrition–P.A.	**1.28 (1.18–1.38)**	**1.52 (1.40–1.64)**	**1.19 (1.09–1.31)**	**1.35 (1.24–1.48)**
Nutrition–Alcohol	**1.30 (1.19–1.42)**	**1.63 (1.50–1.77)**	**1.35 (1.22–1.50)**	**1.44 (1.32–1.57)**
P.A.–Alcohol	**1.57 (1.45–1.71)**	**1.48 (1.36–1.60)**	**1.23 (1.12–1.36)**	**1.19 (1.08–1.30)**
MM3+				
Smoking–Alcohol	**1.30 (1.18–1.43)**	**1.29 (1.17–1.42)**	**1.40 (1.25–1.57)**	**1.38 (1.24–1.54)**
Smoking–Nutrition	**1.25 (1.14–1.38)**	**1.58 (1.42–1.76)**	**1.45 (1.29–1.63)**	**1.69 (1.50–1.91)**
Smoking–P.A.	**1.64 (1.48–1.81)**	**1.49 (1.34–1.65)**	**1.52 (1.35–1.71)**	**1.47 (1.31–1.65)**
Nutrition–P.A.	**1.47 (1.34–1.62)**	**1.74 (1.59–1.90)**	**1.42 (1.27–1.59)**	**1.54 (1.40–1.70)**
Nutrition–Alcohol	**1.39 (1.25–1.55)**	**1.83 (1.66–2.00)**	**1.52 (1.34–1.72)**	**1.60 (1.45–1.77)**
P.A.–Alcohol	**1.94 (1.75–2.14)**	**1.60 (1.45–1.76)**	**1.49 (1.33–1.68)**	**1.23 (1.11–1.37)**
CompxMM				
Smoking–Alcohol	**1.28 (1.16–1.42)**	**1.28 (1.15–1.41)**	**1.35 (1.19–1.53)**	**1.37 (1.22–1.54)**
Smoking–Nutrition	**1.26 (1.14–1.40)**	**1.60 (1.43–1.79)**	**1.46 (1.28–1.66)**	**1.73 (1.52–1.97)**
Smoking–P.A.	**1.67 (1.50–1.85)**	**1.52 (1.36–1.69)**	**1.51 (1.33–1.72)**	**1.50 (1.32–1.70)**
Nutrition–P.A.	**1.56 (1.41–1.73)**	**1.90 (1.73–2.09)**	**1.52 (1.35–1.72)**	**1.69 (1.52–1.88)**
Nutrition–Alcohol	**1.39 (1.23–1.56)**	**1.99 (1.79–2.20)**	**1.53 (1.33–1.75)**	**1.75 (1.57–1.95)**
P.A.–Alcohol	**2.10 (1.87–2.35)**	**1.75 (1.58–1.95)**	**1.60 (1.41–1.82)**	**1.32 (1.18–1.48)**

Emboldened text signifies statistical significance.

**Table 5:** Unadjusted and adjusted odds ratios for incident multimorbidity by aggregated SNAP-HRBs.

SNAP-HRBs Accumulative	Unadjusted OR (95%CI)	Adjusted OR (95%CI) by Age, Sex, and IMD
MM2+		
SNAP 2	**1.47 (1.39–1.56)**	**1.24 (1.16–1.32)**
SNAP 3–4	**1.80 (1.70–1.91)**	**1.52 (1.43–1.63)**
MM3+		
SNAP 2	**1.58 (1.48–1.68)**	**1.29 (1.20–1.39)**
SNAP 3–4	**2.27 (2.12–2.44)**	**1.88 (1.74–2.04)**
CompxMM		
SNAP 2	**1.63 (1.52–1.74)**	**1.31 (1.21–1.42)**
SNAP 3–4	**2.46 (2.27–2.66)**	**1.99 (1.82–2.18)**

Emboldened text signifies statistical significance.

**Table 6:** Unadjusted and adjusted odds ratios for incident multimorbidity by aggregated SNAP-HRBs stratified by sex.

	Odds Ratios for Incident Multimorbidity by Aggregated SNAP-HRBs Stratified by Sex			
SNAP-HRBs Accumulative	Unadjusted OR (95%CI)		Adjusted OR (95%CI) by Age, Sex, and IMD	
	Male	Female	Male	Female
MM2+				
SNAP 2	**1.59 (1.47–1.73)**	**1.40 (1.29–1.51)**	**1.34 (1.22–1.47)**	**1.16 (1.06–1.26)**
SNAP 3–4	**1.79 (1.64–1.95)**	**1.96 (1.80–2.12)**	**1.57 (1.42–1.73)**	**1.53 (1.40–1.68)**
MM3+				
SNAP 2	**1.77 (1.61–1.95)**	**1.45 (1.33–1.58)**	**1.48 (1.33–1.65)**	**1.17 (1.06–1.29)**
SNAP 3–4	**2.34 (2.09–2.61)**	**2.36 (2.15–2.60)**	**2.10 (1.85–2.38)**	**1.81 (1.63–2.01)**
CompxMM				
SNAP 2	**1.81 (1.64–2.01)**	**1.50 (1.36–1.65)**	**1.50 (1.33–1.69)**	**1.18 (1.07–1.32)**
SNAP 3–4	**2.44 (2.16–2.76)**	**2.60 (2.34–2.89)**	**2.17 (1.89–2.49)**	**1.94 (1.73–2.18)**

Emboldened text signifies statistical significance

## Data Availability

Data are unavailable due to privacy or ethical restrictions.

## Abbreviations

The following abbreviations are used in this manuscript:

MM	Multimorbidity
MB	Multibehaviours
SNAP	Smoking, Nutrition, Alcohol, Physical Activity
HRB	Health Risk Behaviours
MBHC	Multiple health behavioural change
MM+2	Multimorbidity of ≥2 morbidities
MM+3	Multimorbidity of ≥3 morbidities
Cmpx MM	Complex multimorbidity (morbidities affecting ≥3 body systems)
CIRS	Cumulative Illness Rating Scale
OR	Odds ratio
CI	Confidence intervals
IMD	Index of Multiple Deprivation
LSOA	Lower Super Output Areas
GP	General Practice
CC	Chronic Conditions
CKD	Chronic Kidney Disease
MS	Multiple Sclerosis
IBS	Irritable Bowel Syndrome
IBD	Inflammatory bowel disease
CLD	Chronic liver disease
COPD	Chronic obstructive pulmonary disease
CHD	Coronary heart disease
Stroke TIA	Stroke and transient ischemic attack
PVD	Peripheral vascular disease
AF	Atrial fibrillation
EHR	Electronic Health Records
CSU	Commissioning Support Unit
